# Accuracy and efficacy of ultrasound‐guided puncture (vs. computed tomography‐guided) in cervical medial branch blocks for cervicogenic pain: A randomized controlled study

**DOI:** 10.1002/ibra.12151

**Published:** 2024-03-17

**Authors:** Jie Tian, Xin‐Yan Li, Yan Yin, Nan Zhao, Hong Xiao, Hui Liu

**Affiliations:** ^1^ Department of Anesthesiology, Sichuan Cancer Hospital & Institute, Sichuan Cancer Center, School of Medicine University of Electronic Science and Technology of China Chengdu China; ^2^ Department of Anesthesiology Southwest Medical University Luzhou Sichuan China; ^3^ Department of Pain Management, West China Hospital Sichuan University Chengdu Sichuan China; ^4^ Department of Anesthesia, Transplant and Surgical Intensive Care Azienda Ospedaliero Universitaria delle Marche Ancona Italy

**Keywords:** cervical medial branch block, cervicogenic pain, computed tomography‐guided, postoperative recovery, ultrasound‐guided in‐plane approach

## Abstract

Cervical medial branch block (CMBB) has been recognized as an effective treatment for cervicogenic pain. Previous studies mostly used ultrasound‐guided out‐of‐plane puncture for CMBB, while this prospective study was designed to investigate the efficacy of ultrasound‐guided in‐plane puncture, specifically focusing on the new target of CMBB for cervical pain. This study includes two parts: the accuracy study (*N* = 15, CMBB was completed by ultrasound and confirmed by computed tomography [CT], in which a good distribution percentage of the analgesic solution was observed) and the efficacy study (*N* = 40, CMBB was completed by ultrasound or CT, while the proportion of pain relief (numerical rating scale) decrease by more than 50% postoperatively was analyzed). The results showed that the good distribution percentage of the analgesic solution was 97.8%. Furthermore, in the early period (30 min and 2 h postoperatively), the proportion of patients with pain relief was lower in the ultrasound group than that in the CT group, especially at 2 h postoperatively (52% vs. 94%). However, at 24 h postoperatively and later, the proportion of patients with pain relief gradually stabilized to about 60%–70%, and lasted for about 2 weeks to 1 month. Therefore, the new target for CMBB, guided by ultrasound in‐plane, offers high visibility and accuracy. A single CMBB performed under ultrasound guidance resulted in pain relief comparable to that of a CT‐guided procedure (1 day to 1 month postoperatively). This study indicated that CMBB guided by ultrasound in‐plane could be regarded as a promising approach for treatment of cervicogenic pain.

## Graphical abstract

Cervical medial branch block (CMBB) has been recognized as an effective treatment for cervicogenic pain. Previous studies mostly used ultrasound‐guided puncture out‐of‐plane for CMBB, while this prospective study was designed to investigate the efficacy of ultrasound‐guided in‐plane puncture, specifically focusing on the new target of CMBB for cervical pain. This study includes two parts: the accuracy study (*N* = 15, CMBB was completed by ultrasound and confirmed by computed tomography [CT], in which a good distribution percentage of the analgesic solution was observed) and the efficacy study (*N* = 40, CMBB was completed by ultrasound or CT, while the proportion of pain relief (numerical rating scale) decrease by more than 50% postoperatively was analyzed). The results showed that the good distribution percentage of analgesic solution was 97.8%. This study indicated that CMBB guided by ultrasound in‐plane could be regarded as a promising approach for cervicogenic pain.

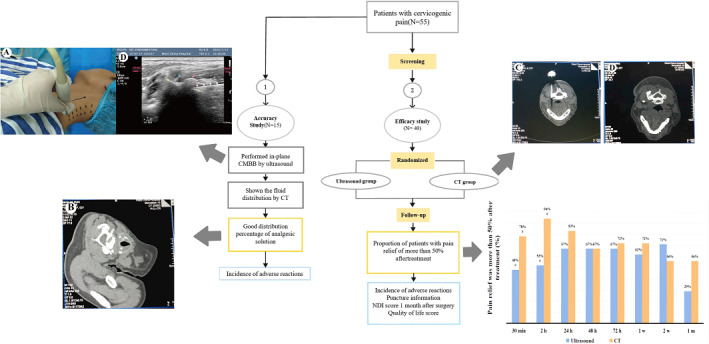

## 1 INTRODUCTION

**Figure 1:**
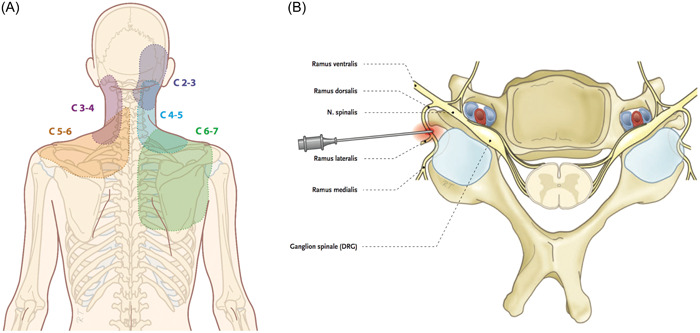
Anatomy of cervicogenic pain. (A) Posterior medial branch of each cervical spinal nerve innervating cutaneous sensory areas. Image obtained from Rogier Trompert Medical Art. (B) New target located at the superior border of the cervical transverse process with the superior articular process migration. Image obtained from Rogier Trompert Medical Art. [Color figure can be viewed at wileyonlinelibrary.com]

Cervicogenic pain is a common ailment in modern society, which is significantly related to stress, work‐related factors, psychological disorders, and other factors.^1^, ^2^ It has also been noted that pain at a young age can persist into adulthood and have a lifelong impact.^3^ The etiology of cervicogenic pain is complex, and the vertebral body, intervertebral disc, Luschka's joint, facet joints, muscles, and ligaments may be the cause of pain.^4^ The entrapment of the posterior branch and its branches of the cervical spinal nerve and the structure disturbance of the small joint innervated are the main causes of cervicogenic neck pain^2^, ^5^ (Figure 1A). Therefore, cervical medial branch block (CMBB) has been recognized as an effective treatment for cervicogenic pain in recent years,^6^, ^7^ in which a local anesthetic is injected around the medial branches to block or reduce pain caused by pathological factors.

In the past, CMBB was performed blind, which had the advantages of simplicity and convenience, but the accuracy was poor, and there was an increased risk of injury to cervical blood vessels, nerves, soft tissues, and even the spinal cord. In order to further improve the accuracy of CMBB, it is often performed under the guidance of computed tomography (CT), which is an accurate method of localization. However, it is difficult to avoid radiation damage during puncture scanning, and the operation is limited by the CT scanner. With the increasing use of ultrasound in the field of pain, it has become an option for minimally invasive interventions. The biggest advantage is that it realizes real‐time visualization of the target structure and its surrounding tissues, the puncture path of the puncture needle, and distribution of the medication during the puncture process, which increases the accuracy of the puncture and injection process.^8^, ^9^ At the same time, it avoids radiation, and is easy to carry, because of which bedside operations can be carried out.^10^

It is worth noting that previous studies mostly used ultrasound‐guided out‐of‐plane puncture for CMBB. In this approach, the needle insertion site was located behind the vertebral artery plane and the needle was inserted from anteriorly outward to posteriorly medial, which was close to the vertebral artery. This may result in vertebral artery injury and bleeding. In addition, there exist uncertainties in finding the highlighted echo point indicating the tip of the needle on ultrasound images.^11^, ^12^ Therefore, it is important to explore a new target for CMBB, particularly utilizing ultrasound in‐plane. Most of the posterior medial branch in the cervical spinal nerve is located in the midpoint of the adjacent upper joint vertex and the upper one‐third of the midline of the joint column.^13^ Combined with the target of the CMBB under CT guidance, this study found that the new target was located at the upper edge of the transverse process and the upper joint of the corresponding cervical segment^6^, ^11^ (Figure 1B). Compared with the target selected for out‐of‐plane blockade of this nerve (lumbar portion of the articular column), the new target is closer to the proximal end of the nerve.^14^ Previous case reports have also indicated the advantages of the in‐plane approach ultrasound‐guided cervical nerve block, but no further systematic comparisons and discussions have been conducted.^15^ Therefore, in this study, the accuracy of CMBB under the ultrasound guidance in‐plane approach was confirmed, and the efficacy and safety of ultrasound‐guided were also evaluated by comparing with classical CT‐guided CMBB in patients with cervicogenic pain through a randomized controlled trial. This may provide a clinical basis for the realization of ultrasound‐guided in‐plane posterior medial cervical spinal nerve block in the field of neck pain treatment.

## 2 METHODS

### 2.1 Patients and grouping

This study was approved by the Ethics Committee of West China Hospital, Sichuan University (No. 2013 (61)). All procedures in this study were performed according to the relevant guidelines and regulations. Patients with cervicogenic pain who visited the Pain Department of West China Hospital of Sichuan University between July 2015 and December 2016 were included in the screening.

Inclusion criteria were as follows: (1) age range from 18 to 80 years; (2) patients with pain in the area of distribution of the posterior medial branch of the spinal nerve at the cervical vertebra (C2‐6) (Figure 1A); (3) area of pain that was unilateral and covered three areas where the spinal nerves were distributed; (4) patients with cervicogenic pain lasting more than 3 months with poor treatment effect; (5) average numeric rating scale (NRS) (a range of 0–10, with a score of 0 indicating no pain and a score of 10 indicating excruciating pain) ≥4; and (6) no history of invasive treatment or surgery on the neck in the last 3 m.

Exclusion criteria were as follows: (1) patients with severe cardiopulmonary disease; (2) patients with spinal deformities; (3) patients who are allergic to local anesthetics or glucocorticoid; (4) patients with coagulation dysfunction; (5) patients with skin infection at the puncture site; (6) patients with opioid addiction; (7) patients with psycho‐behavioral abnormalities and severe anxiety and/or depression; (8) pregnant and breastfeeding women; and (9) patients with other chronic diseases that could interfere with pain assessment.

**Figure 2:**
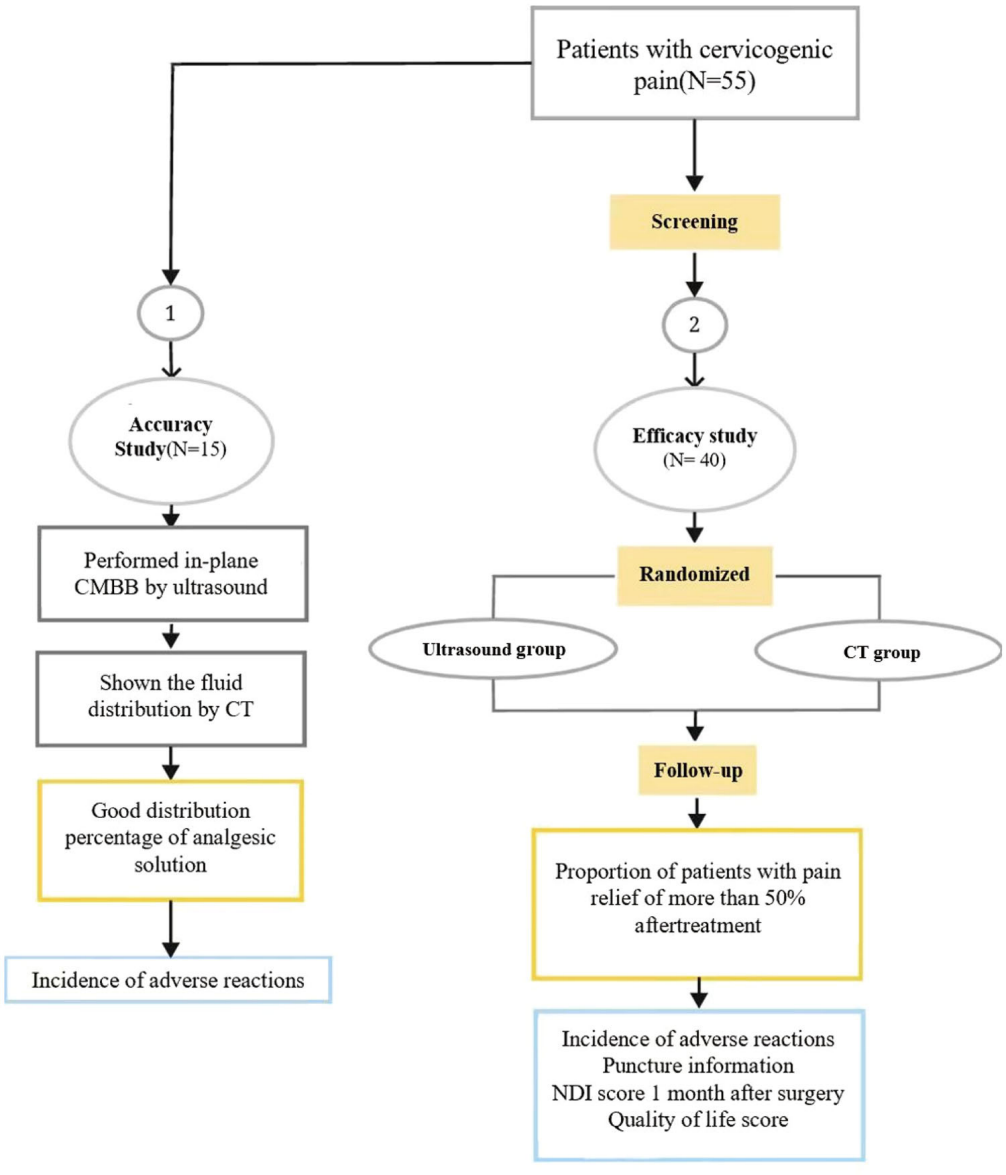
Research program flow. CMBB, cervical medial branch block; CT, computed tomography; *N*, number(s); NDI, neck disability index. [Color figure can be viewed at wileyonlinelibrary.com]

During the screening period, patients' basic information (including gender, age, height, weight, body mass index [BMI]), and severity of disease (pretreatment NRS score, pain duration, neck disability index [NDI] score, and quality‐of‐life score) was obtained. Then, all included patients were randomly divided into an ultrasound group or a CT group using random numbers. The relevant operations and observations were carried out according to the groups of patients (Figure 2).

### 2.2 Main materials and instrument

The main materials and instruments used in this study included the Philips CX50 ultrasound machine (Philips) and Siemens 16‐row spiral CT (Siemens).

### 2.3 Selection of targets

The upper edge of the transverse process of the corresponding cervical vertebral segment and the superior articular process migration were localized (Figure 1B).

#### 2.3.1 Ultrasound‐guided in‐plane CMBB

**Figure 3:**
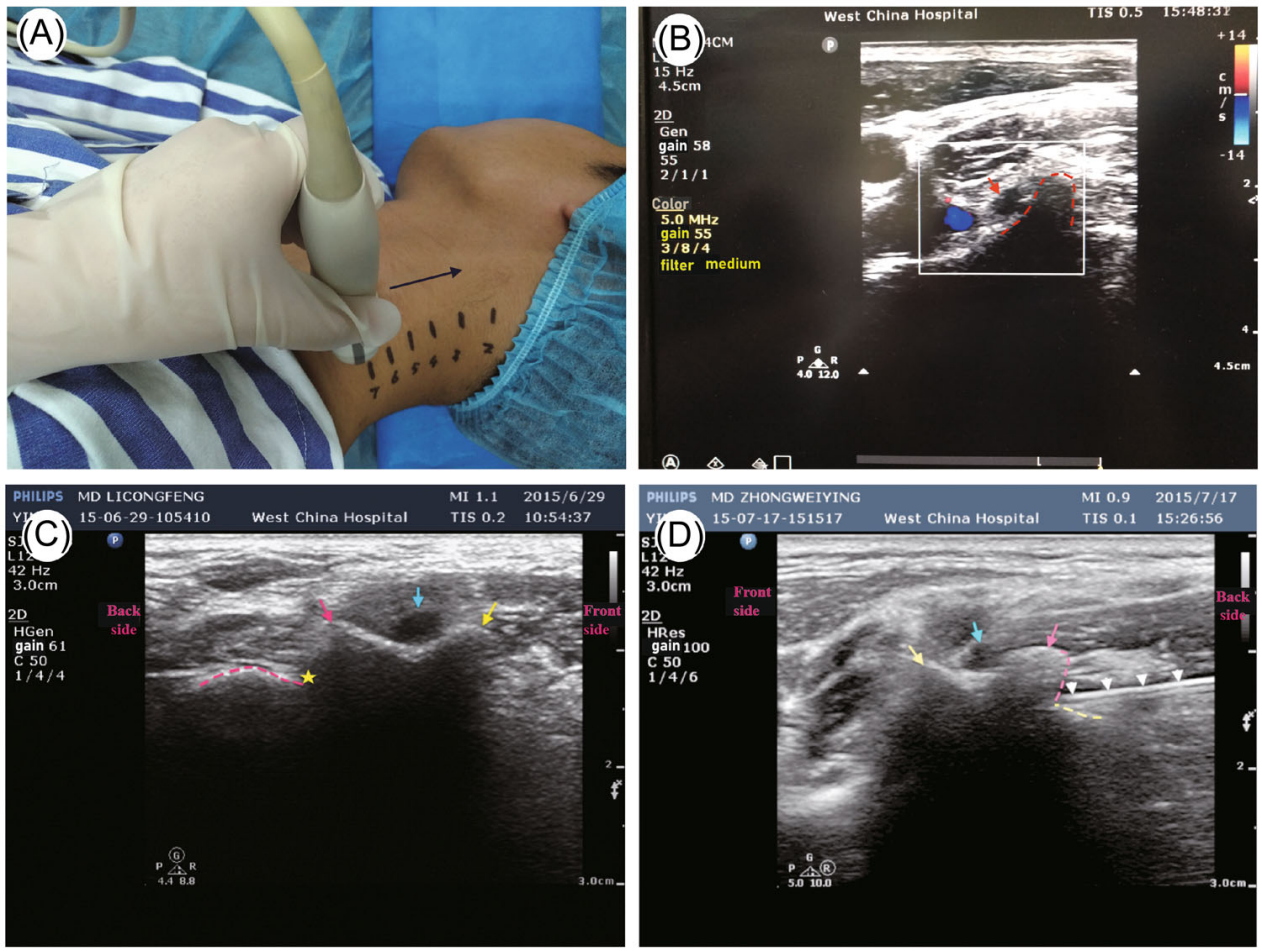
In‐plane cervical medial branch block by ultrasound. (A) Positioning during ultrasonic imaging. (B) Image of C7 by ultrasound: the red dotted line shows the C7 transverse process, the red arrow shows the C7 nerve root, and the blood flow signal shows the vertebral artery. (C) Image of C6 by ultrasound: yellow arrows show the pretransverse nodes, the red arrows show posttransverse nodules, blue arrows show C6 nerve roots, the red dotted line shows the superior articular process, and the yellow asterisk shows the new target site. (D) Plane of the new target under ultrasound guidance: white arrows show the stem and tip of the needle. The red dotted line shows the upper edge of the posttransverse tubercle, the yellow dotted line shows the superior articular process, and the migration is the target location. Yellow arrows show pretransverse nodes, red arrows show posttransverse nodes, and blue arrows show nerve roots. C, cervical. [Color figure can be viewed at wileyonlinelibrary.com]

The patients were placed in lateral recumbency on the operating platform, with the affected side of the neck facing upward and a thin pillow under the head. The operator selected a high‐frequency probe coated with a coupling agent, and performed a cross‐sectional scan of the cervical spine (Figure 3A). The detailed steps were as follows: First, the operator located the transverse process of the seventh cervical vertebra (C7), which had only posterior nodules, and the ultrasound image was similar to the back of a chair (Figure 3B), then slowly moved the probe to the cephalic side, till the C6 transverse process with visible anterior and posterior nodules, and the ultrasound image showed a hump‐like shape (Figure 3C). Then, the operator moved the probe to the upper margin of the C6 transverse process, and fine‐tuned it to the lower transverse process, till the upper joint synchondrosis were visible, where the target point was precisely located. At this point, the upper edge of the transverse process and the superior articular process were visible at the same time (Figure 3D), which was the plane of the target. Because the ultrasound image of the transverse process of the upper cervical spine was similar to that of the C6 transverse process, the probe can be moved to the side of the head to locate the target planes of the upper cervical spine segments in turn, and the corresponding markings were made on the skin.

#### 2.3.2 CT‐guided CMBB

The patients were placed in a prone position on the operating platform of the CT, with a thin pillow placed on the forehead. The whole back of the neck was exposed, and the first CT localization scan was performed after sticking a radiopaque marker on the affected side. The needle path was designed in the localization phase, and the laser positioning function of CT was used to mark the needle points on the patient's skin surface.

### 2.4 Operation and outcomes

#### 2.4.1 Accuracy of CMBB under the ultrasound guidance in‐plane approach

##### Operation

**Figure 4:**
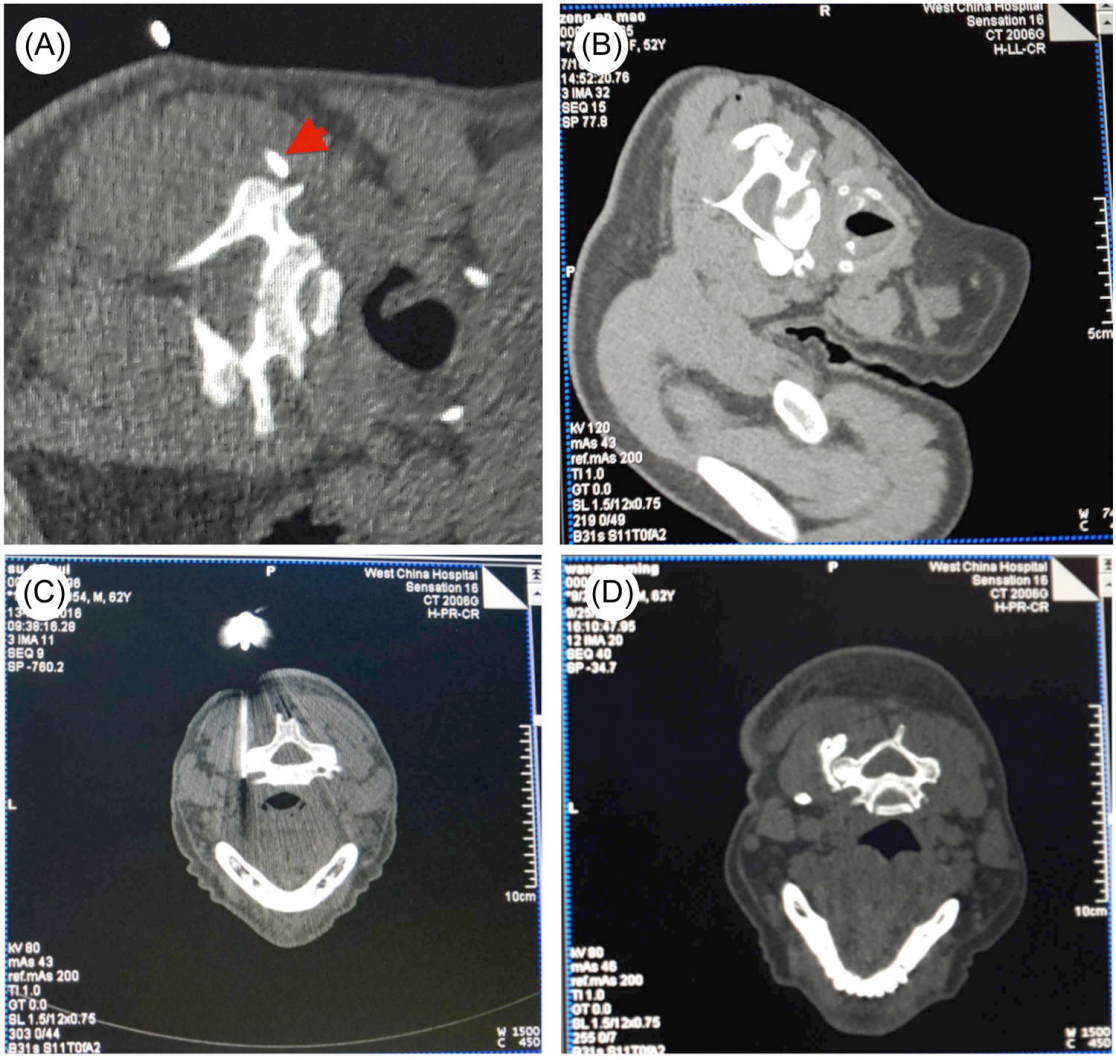
Cervical medial branch block by computed tomography (CT). (A) CT scan verifying the tip of the puncture needle: The tip of the needle has successfully reached the target site; the red arrow shows the tip of the needle. (B) Drug diffusion under CT scan: good diffusion of the drug is illustrated. (C) CT‐guided puncture tip successfully reaching the target site. (D) Drug diffusion under CT‐guided cervical medial branch block. [Color figure can be viewed at wileyonlinelibrary.com]

According to the area of pain distribution of the patients, three unilateral cervical spinal nerve posterior medial branch puncture segments were identified for each patient (Figure 1A). After the patients were admitted to the operation room, the intravenous access was opened and routine cardiac monitoring was performed. For each segment, after determining the target of CMBB, disinfection was carried out with the puncture site as the center. The high‐frequency probe was coated with medical ultrasonic couplant and placed in a sterile surgical film, which was subsequently placed at the intended puncture site, and color Doppler was used to identify the blood vessels around the puncture path and determine the needle insertion path. After administration of local anesthesia with 1% lidocaine, an ultrasound‐guided in‐plane injection (22 G puncture needle) was administered when the tip reached the target (Figure 3D). Once the puncture was completed, another doctor performed a CT scan to confirm the location of the needle tip as soon as possible. The scanning thickness was 1.5 mm, and the distance between the needle tip and the target was defined as no more than 3 mm (Figure 4A). If the first puncture was unsuccessful, the position of the puncture needle was adjusted again under ultrasound guidance, and each adjustment needed to be confirmed by CT scan. After successful puncture was confirmed, 1 mL of analgesic solution was injected into each segment (Formulation of analgesic solution: 5 mL = diprospan 0.5 mL + VitB_12_ 1 mL + 2% lidocaine 1 mL + 1% ropivacaine 1 mL + contrast medium 1 mL + 0.9% normal saline 0.5 mL). CT scan was performed again to observe the distribution of liquid medicine (Figure 4B). At the end of the procedure, the puncture site was disinfected, followed by a sterile application.

##### Outcomes

The results were evaluated on the basis of two outcomes: primary outcome and secondary outcome. The former was indicated by good distribution percentage of the analgesic solution, while the latter was indicated by the incidence rate of adverse reactions, such as nerve damage, paresthesia, nausea and vomiting, puncture into blood, dizziness, and so on. For primary outcome, there are three levels of distribution of pain relief solutions, including “ideal,” “good,” and “poor,” whose criteria are as follows: When the analgesic solution was distributed between the upper edge of the transverse process and the superior articular process, surrounding the articular column backward (Figure 4B), it was regarded as “ideal”; if the liquid was distributed between the upper edge of the transverse process and the superior articular process without wrapping backward around the articular column, it was evaluated as “good.” However, when the liquid was not distributed between the superior border of the transverse process and the superior articular process, it was regarded as “poor.”

#### 2.4.2 Efficacy of CMBB using different methods (ultrasound‐guided using the in‐plane approach vs. CT‐guided)

##### Operation

The operation procedure of the ultrasound group was the same as described in previous paragraph. The puncture time was defined as the time starting from the probe touching the patient's skin and terminating after completing the injection, with the disinfection time excluded.

For the CT group, after the patient was admitted to the operation room, intravenous access was opened and routine cardiac monitoring was performed. The whole back of the patient's neck was exposed, and the first CT localization scan was performed after sticking a radiopaque marker on the affected side, and the timing was started. Following routine disinfection and towel draping, local anesthesia was administered using 1% lidocaine. Subsequently, a No. 7 puncture needle was inserted along the predetermined route. Each needle placement was verified by another physician using CT. With a scanning thickness of 1.5 mm, the distance between the tip of the needle and the target point within 3 mm was regarded as a successful puncture (Figure 4C). If the initial puncture attempt was unsuccessful, the needle position was adjusted, with each adjustment confirmed by CT scanning. After confirming the success of the puncture, each segment was injected with 1 mL of analgesic solution (same as the ultrasound group), and the timing was terminated (the sterilization time was not counted during the period). Then, the CT scan was performed again to observe the distribution of the medicinal solution (Figure 4D). Finally, the puncture site was sterilized and a sterile dressing was applied.

##### Outcomes

The primary outcome included the proportion of patients with pain relief of more than 50% after 48 h of treatment compared with before treatment (percentage of pain relief at 48 h).

Secondary outcomes included (1) percentage of pain relief at 30 min, 2 h, 24 h, 72 h, 1 week, 2 weeks, and 1 month postoperatively; (2) the rate of adverse reactions (including nerve damage, paresthesia, nausea and vomiting, puncture into blood, dizziness, etc.); (3) times of each puncture and operation time; (4) NDI score 1 month after surgery (this scale is used to assess cervical spine dysfunction. The questionnaire consists of 10 questions, with six choices for each question, and the highest score is 5 points. The highest score of the questionnaire was 50 points and the lowest score was 0 points. The higher the score, the more serious the cervical spine dysfunction); and (5) quality‐of‐life score (by brief pain inventory, BPI. It consists of seven items that are assessed on a scale of 0 to 10 based on pain, including daily living, mood, ability to walk, ability to function, relationships with others, sleep, and enjoyment of life. The overall score is 70, and the higher the score, the greater the impact of pain on their quality of life.) 1 month after surgery.

### 2.5 Randomization and blind

The randomization numbers were acquired through a randomized table. Subjects who did not meet the study inclusion criteria or did not meet any exclusion criteria were not eligible for randomization to the study. In the efficacy research, the operator was not informed of the method of operation until the moment before the operation was carried out. All puncture procedures were performed by the same experienced physician and evaluated by another physician who was not aware of the study during the operation. Other data collectors were only involved in obtaining the records before and after the operation, recording the basic characteristics of the patients, the NRS scores before and after the operation, the NDI scores, and quality‐of‐life scores.

### 2.6 Sample size

The sample size was calculated using SPSS 11.0. For assessment of accuracy, the degree of drug diffusion under CT was used to evaluate the accuracy of selection of new targets. Sample sizes of 15 for ultrasound guidance achieved 86% power to detect a non‐inferiority margin difference. The reference group (CT guidance) proportion was 0.99. The test statistic used in this study was the one‐sided *Z* test (pooled). The significance level of the test was set at 0.025. Therefore, for the assessment of accuracy of in‐plane CMBB under ultrasound guidance, was decided to include 15 patients for a preliminary experiment. For the assessment of efficacy of CMBB, according to previous studies,^16^ about 85% of patients reported pain relief of more than 50%. The test efficiency is at 80% with a target significance level of 0.05. The non‐inferiority margin was set at 10% for this clinical trial. Therefore, about 16 patients were required for each group. Considering potential loss to follow‐up of 10% of patients, a minimum of 18 patients were ultimately required in each group.

### 2.7 Statistical analysis

All data were entered into SPSS 21.0 for statistical analysis. Expectation‐Maximization algorithm (EM) missing value analysis was used to supplement the missing data. Measurement data were analyzed using an independent *t*‐test (normal distribution data) or the Mann–Whitney *U* test (non‐normal distribution data) and expressed as mean ± standard deviation (SD) (normal distribution data) or median (P 25, P 75) (non‐normal distribution data). NRS was analyzed and compared using repeated measurement variance. Qualitative data were analyzed using the *χ* ^2^ test and expressed as numbers (percentages). *p < 0.05 was considered to indicate statistical significance.

## 3 RESULTS

Between June 2015 and December 2016, a total of 55 patients with cervicogenic pain were included on the basis of the inclusion and exclusion criteria. The assessment of accuracy of in‐plane CMBB under ultrasound guidance was performed in 15 of these patients. The assessment of efficacy of CMBB using different methods of guidance (ultrasound group: *N* = 21; CT group: *N* = 19) was conducted in the other 40 patients. Only one patient in the CT group was lost to follow‐up; therefore, information from 39 patients was analyzed in the assessment of efficacy.

### 3.1 Accuracy analysis

#### 3.1.1 Basic information

The average age of the 15 study subjects (11 males and four females) was 51.5 ± 10.8 years, the average duration of pain was 24 m, and a total of 45 posterior medial branches of cervical spinal nerves were punctured, of which the percentage of punctures in each segment was as follows: C3: 20.0% (*N* = 9), C4: 33.3% (*N* = 15), C5: 33.3% (*N* = 15), and C6: 13.3% (*N* = 6). The rate of new target visualization under ultrasound of 45 posterior medial branches of cervical spinal nerves was 100%.

#### 3.1.2 Outcomes

Of the 45 puncture sites, the overall success rate of one‐time puncture was 71.1% (32/45). The success rates of one‐time puncture for each segment were as follows: C3: 55.6% (5/9); C4: 60.0% (9/15); C5: 86.7% (13/15); and C6: 83.3% (5/6). The one‐time success rate of puncture in the low cervical segment (such as C5‐6) was higher than that of the high cervical segment (such as C3‐4). After the analgesic solution was administered, about 80.0% of the patients (36/45) had ideal fluid distribution, 17.8% of the patients (8/45) had good fluid distribution, and 2.2% of the patients (1/45) had poor fluid distribution. The overall ratio of ideal and good distribution of analgesic solution was 97.8%. In all punctured segments, there was no case of intravascular injection or diffusion of the drug into the intervertebral foramen. There were two cases of postoperative dizziness and one case of postoperative nausea (relieved after 30 min), and no other adverse reactions were found.

### 3.2 Efficacy analysis

#### 3.2.1 Basic information

Each patient had three puncture segments of the posterior medial branch of the cervical spinal nerve on one side based on their pain distribution region (Figure 1A). There was no significant difference between the ultrasound group and the CT group in the patients' basic characteristics (including gender, age, height, weight, BMI) and severity of disease (pretreatment NRS score, pain duration, NDI score, and quality‐of‐life score) (all *p* > 0.05, Table 1).

**Table 1:** Comparison of basic information.

		Ultrasound group	CT group	*p* Value
		(*N* = 21)	(*N* = 18)	
Gender, *n* (%)	Male	8 (38)	8 (44)	0.688
	Female	13 (62)	10 (56)	
Age, years, *M* ± SD		43.2 ± 10.6	47.9 ± 12.5	0.212
Height, cm, *M* ± SD		162.4 ± 7.3	163.2 ± 7.7	0.741
Weight, kg, *M* ± SD		58.8 ± 7.6	62.2 ± 9.7	0.228
BMI, kg/m^2^, *M* ± SD		22.3 ± 2.3	23.3 ± 2.6	0.21
Duration of pain before treatment, months, Md (IQR)		24 (5,36)	30 (12,86)	0.642
Segment of puncture, *n* (%)	C2	6 (9.5)	10 (18.5)	/
	C3	12 (19.0)	12 (22.2)	/
	C4	21 (33.3)	18 (33.3)	/
	C5	15 (23.8)	8 (14.8)	/
	C6	9 (14.3)	6 (11.1)	/

*Note*: /, The puncture points of patients were selected according to their condition, so no comparison was made.

Abbreviations: BMI, body mass index; C, cervical; CT, computed tomography; IQR, interquartile range; *n*, number; *M*, mean; Md, median; SD, standard deviation.

#### 3.2.2 Outcomes

The average number of punctures in the ultrasound group (4.0 ± 1.1 times) was significantly lower than that in the CT group (8.0 ± 2.3 times). Besides, the operation time was shorter in the ultrasound group (26.9 ± 9.8 min, vs. 32.1 ± 8.6 min; *p* > 0.05).

**Figure 5:**
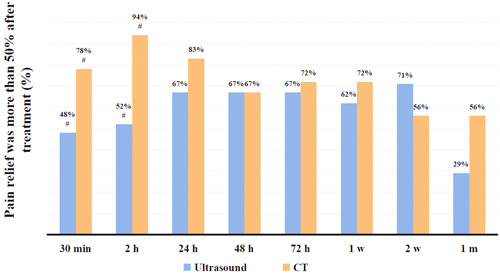
Pain relief was more than 50% after treatment (%). CT, computed tomography; m, month(s); w, week(s). ^#^Comparison between the two groups, *p* < 0.05. [Color figure can be viewed at wileyonlinelibrary.com]

The NRS scores of the two groups were lower than the preoperative scores at each observation point after surgery (Table 2). The proportions of patients with pain relief at 48 h postoperatively in the two groups were 67%, and the difference was not statistically significant. Interestingly, in the early period after CMBB (30 min and 2 h postoperatively), the proportion of patients with pain relief was lower in the ultrasound group than that in the CT group, especially at 2 h postoperatively (52% vs. 94%). After 24 h, the pain relief rate in both groups gradually stabilized to about 60%–70%. The whole pain relief condition may last for about 2 weeks to 1 month (Figure 5). There were five patients with postoperative dizziness (three cases in the ultrasound group and two cases in the CT group), and this was relieved in all of them after 30 min. There was no significant difference between the two groups. No other adverse reactions were observed.

**Table 2:** Comparison of NRS scores before and after treatment.

*M* ± SD	Ultrasound group (*N* = 21)	CT group (*N* = 18)	*p* Value
Pre‐	4.8 ± 1.7	4.9 ± 1.6	0.647
Post‐30 min	2.4 ± 1.9* ^,^ ^#^	1.2 ± 0.7* ^,^ ^#^	0.009
Post‐2 h	2.2 ± 1.7* ^,^ ^#^	0.8 ± 0.5* ^,^ ^#^	<0.001
Post‐24 h	2.0 ± 1.8*	1.3 ± 0.8*	0.054
Post‐48 h	2.0 ± 1.6*	2.3 ± 2.0*	0.963
Post‐72 h	2.1 ± 1.7*	2.5 ± 1.7*	0.748
Post‐1 week	2.6 ± 1.7*	2.3 ± 1.6*	0.694
Post‐2 weeks	2.2 ± 1.8*	2.4 ± 1.9*	0.073
Post‐1 month	3.4 ± 1.9*	2.3 ± 2.0*	0.355

*Note*: NRS was analyzed and compared using repeated measurement variance. The main effect of the number of measurements was significant, *F* = 33.831, *p* < 0.001, *η* ^*2*^ = 0.857. The interaction effect of the number of measurements and the group size was significant, *F* = 3.709, *p* = 0.002, *η* ^*2*^ = 0.397. Machly *W* = 0.046, *p* < 0.001. By Greenhouse–Geisser correction, the main effect of the number of measurements was significant (*p* < 0.001), and the interaction effect of the number of measurements and the group size was significant (*p* = 0.003).

Abbreviations: CT, computed tomography, *M*, mean; *N*, number; NRS, numerical rating scale; SD, standard deviation.

* Compared with before treatment, *p* < 0.05.

^#^ Comparison between the two groups, *p* < 0.05.

The NDI scores of the two groups of patients at 1 month after surgery were lower than those before surgery (Pre: 15.0 ± 5.7 [ultrasound] vs. 10.3 ± 5.4 [CT]; Post: 11.3 ± 6.0 ve. 8.2 ± 5.4). There was a statistically significant difference between before and after, but there was no statistical difference between the two groups. The total quality‐of‐life scores before surgery and 1 month after surgery were 25.0 ± 12.1 and 13.4 ± 11.0 in the ultrasound group and 22.6 ± 12.1 and 10.2 ± 8.8 in the CT group, respectively. There was no statistical difference between the two groups.

## 4 DISCUSSION

### 4.1 Accuracy comparison

In the present study, the visibility of the new target point in the 45 puncture segments under ultrasound was 100%, indicating certain feasibility in the clinical operation. The one‐time success rate of CMBB by the ultrasound‐guided in‐plane approach was 71.1%, which was similar to that of previously reported ultrasound‐guided out‐of‐plane nerve blocks.^14^, ^17^ Meanwhile, the one‐time success rate of puncture in the low cervical segment was higher than that of the high cervical segment. This may be attributed to the increasingly shorter cervical transverse processes in high cervical spine, as well as obstruction of the mandible to the placement of the probe, which resulted in poorer visualization of the ultrasound image in the high cervical segment and the difficulty of puncture. According to the study of Manchikanti et al.,^18^ 1 mL of analgesic solution was injected into each segment, and the distribution of the drug solution was observed through CT. Our study found that the overall ratio of good distribution of the analgesic solution was 97.8%, of which 80.0% of the analgesic solution was distributed between the upper edge of the transverse process and the superior articular process and encircled the articular column posteriorly. That is to say, the drug was distributed in complete alignment along the posterior medial branch of the cervical spinal nerve. In addition, 17.8% of the analgesic solution was distributed between the superior border of the transverse process and the superior articular process. Although the drug solution did not encircle the articular column backward, the drug solution was sufficient to achieve a blocking effect on the proximal part of the nerve.^19^ A previous study reported that the actual position of the posterior medial branch of the cervical spinal nerve under ultrasound in the long‐axis view of the spine had a deviation of 1–2.2 mm, comparing the bony target point of the nerve block under CT.^12^ Covering the whole joint column with liquid medicine can better avoid the block insufficiency caused by nerve deformation and variation. Therefore, irrespective of the anatomical characteristics,^13^ ultrasound imaging,^20^ or the verification results of CT,^21^, ^22^ it is feasible to perform CMBB in plane under ultrasound guidance, and the puncture accuracy is high.

In addition, as ultrasound‐guided in‐plane needle insertion enables full visualization of the puncture needle, it can prevent severe and irreversible complications such as spinal cord infarction caused by intravascular injections to a certain extent.^23^, ^24^ Cadaveric studies have shown that the posterior medial branch of the spinal nerves of each segment of the C3‐8 is accompanied by 1–2 blood vessels. The incidence of intravascular injections was 3.9% in cervical spinal nerve block under CT, which is higher than that of the thoracic and lumbar segments.^25^ Due to the contrast medium, there was no case of intravascular injection of the drug solution in this study, but two patients had postoperative dizziness. The reason for this may be related to the fact that the target point is close to the intervertebral foramen, which is rich in peripheral blood vessels, and rapid absorption of the drug solution occurred in a short period of time. Meanwhile, there was no case of intravertebral foraminal diffusion in this study, which prevented the occurrence of radicular block. The reason for this may be that after excluding the factor of sample size, when the needle was inserted from the back to the front, the angle of the needle was smaller, which was more in line with the anatomical shape of the posterior medial branch of the cervical spinal nerves, and the liquid was more likely to be distributed along the nerves. However, further clinical studies are needed to determine whether the difference in needle angle affects the imaging distribution of the drug. In conclusion, ultrasound‐guided in‐plane posterior medial branch block of the cervical spinal nerve has a high degree of safety.

### 4.2 Efficacy comparison

Posterior medial cervical spinal nerve blocks were initially used to diagnose pain of facet joint origin and to predict the efficacy of radiofrequency destruction of the posterior medial branch of the cervical spinal nerve, but multiple repeated blocks can be effective in treating cervicogenic pain.^26^ A previous study demonstrated that more than 85% of patients had more than 50% pain relief after 2 years of repeated cervical spinal nerve blocks. Therefore, posterior medial cervical spinal nerve block has good efficacy for patients with cervicogenic pain and is recommended by the guidelines.^16^ In this study, the target point for ultrasound‐guided in‐plane CMBB is the same as that of CT‐guided block. The CT group was selected as a positive control, which could avoid bias to some extent and enabled better evaluation of the effectiveness of ultrasound‐guided in‐plane posterior medial branch block of the cervical spinal nerve.

In this study, the pain levels of both groups of patients at each observation point after surgery were less than those before surgery. However, the NRS scores of the CT group at 30 min and 2 h after surgery were lower than that in the ultrasound group. At the same time, the proportion of patients in the CT group who had more than 50% pain relief 2 h after surgery was as high as 94%, which is significantly higher than that in the ultrasound group. This may be due to the fact that patients in the CT group had a shorter vertical skin needle access path than that in the ultrasound group with an oblique marching needle in this study. This caused more tissue damage in the ultrasound group, leading to more acute inflammatory pain, which partly interfered with the patients' evaluation of the treatment outcome. In addition, it has been found that when the needle is inserted vertically, the length of contact between the needle and the nerve is longer.^13^ This may be more conducive to the infiltration of the nerve by the drug solution in the short term and accelerate the speed of pain relief. However, whether the needle insertion angle can affect the diffusion of the medicinal solution and produce obvious differences in short‐term efficacy requires clinical research with a large sample size. In this study, the rate of significant pain relief (the proportion of patients with more than 50% relief) stabilized at about 1 day after surgery (about 67% to 83%) and lasted from about 2 weeks to 1 month. The results of the study coincide with the time of clinical re‐treatment.

Although the puncture targets selected for the ultrasound group and the CT group were the same, the average number of punctures in the ultrasound group was less, and the one‐time puncture success rate was higher. Correspondingly, the surgical time was shorter in the ultrasound group. This is similar to the results of previous studies that directly compared two imaging intervention methods.^18^, ^27^ This is probably because with ultrasound, real‐time visualization of the target structure and its surrounding tissues, the puncture needle puncture path, and diffusion of the drug solution during the puncture process is possible. In addition, CT guidance is operated in cross section, which is similar to out‐of‐plane ultrasonic guidance, and accurate visualization of the needle path may not be possible.

One month after CMBB by different approaches, the NDI scores and quality‐of‐life scores of the patients were significantly improved compared with those before surgery, and there was no significant difference between the groups. Ultrasound‐guided in‐plane CMBB can not only achieve similar effects to CT guidance in pain relief but can also effectively improve cervical spine function and quality of life in patients with cervicogenic pain. In this study, patients with cervical pain had no obvious organic injury. Therefore, pain control can improve the functional activity of the neck in patients.^28^ There have been a lot of studies ^29^, ^30^ on the impact of chronic pain on the quality of life of patients. The improvement of pain can improve the quality of life of patients, which indicates that ultrasound‐guided in‐plane CMBB is of great practical value.

### 4.3 Strengths and limitations

In previous studies, CMBB was operated outside the plane by CT or ultrasound. Because the “real‐time guidance” in the operation process can be realized in the plane, safety is significantly higher than that outside the plane. Therefore, the first innovation of this study was that a new operational target was selected in this study to realize in‐plane CMBB guided by ultrasound. Since this target has not been mentioned in the relevant literature before, the design of this study was divided into two parts. In the first part, the location selection of the new target was accurate through the degree of diffusion of analgesics. In the second step, the effectiveness of ultrasound‐guided treatment after the new target operation was determined on the basis of aspects of pain relief rate, NDI score, and quality‐of‐life assessment before and after treatment by comparing with CT guidance. However, the collecting operation procedure data has a relationship with the operator's procedure abilities, and the results of this study may only reflect the experience level of the operator, and there may be certain limitations in its generalizability. Furthermore, this study only evaluated the short‐term efficacy of ultrasound‐guided in‐plane CMBB, and the long‐term efficacy should be studied further. In the future, we will expand the sample size and follow‐up duration, and evaluate postoperative recovery in terms of multiple factors, including patient comfort, independence, emotions, pain, etc.

## 5 CONCLUSION

The new target of CMBB by the ultrasound‐guided in‐plane approach has high visibility, accuracy, and safety. A single CMBB by the ultrasound‐guided in‐plane approach resulted in a similar degree of pain relief as the CT‐guided approach (1 day to 1 month postoperatively), with recovery of neck function and improvement in quality of life 1 month after surgery.

## AUTHOR CONTRIBUTIONS

All authors are responsible for the data, analysis and interpretation, and conduct of the study, and had full access to all data. Jie Tian set the data criteria and performed the data collection, initially analyzed the data, and wrote the manuscript. Xin‐Yan Li was involved in further analysis of the data, and wrote the article. Nan Zhao was involved in data collection. Yan Yin participated in the implementation of treatment. Hong Xiao directed the project and revised the article. Hui Liu provided and revised the data analysis, made comments, and revised the manuscript. In addition, all authors' personal communications have been cited with permission.

## CONFLICT OF INTEREST STATEMENT

The authors declare no conflicts of interest.

## ETHICS STATEMENT

This study was conducted in accordance with Good Clinical Practice Guidelines, as well as the principles of the Declaration of Helsinki, and was approved by the local ethics committee (the Ethics Committee of West China Hospital, Sichuan University, No. 2013(61)). Written informed consent was obtained from all the patients included in this study.

## Data Availability

The data that support the findings of this study are available from the corresponding author upon reasonable request.
